# Poor nutritional status based on controlling nutritional status score and nutritional risk index may be related to the occurrence of complications after laparoscopic treatment in patients with gastrointestinal tumors aged no more than 60 years

**DOI:** 10.3389/fonc.2025.1723703

**Published:** 2025-12-15

**Authors:** Xueyong Deng, Zhiwang Li, Lv Ye, Chengkun Yang

**Affiliations:** 1 Department of Gastrointestinal Surgery, Meizhou People’s Hospital, Meizhou, China; 2 Department of Psychology, Meizhou Third People’s Hospital, Meizhou, China

**Keywords:** controlled nutritional status, gastrointestinal tumor, laparoscopic treatment, nutritional risk index, postoperative complication

## Abstract

**Background:**

Nutritional status is related to the postoperative complications in gastrointestinal tumors. Early-onset and late-onset tumors have differences in molecular characteristics and clinical manifestations. The purpose of this study is to explore the relationship between preoperative nutritional assessment indices (prognostic nutritional index (PNI), nutritional risk index (NRI), and controlled nutritional status (CONUT) score) and the occurrence of complications after laparoscopic treatment in patients with gastrointestinal tumors aged ≤60 years.

**Methods:**

1405 patients with gastrointestinal tumors aged ≤60 years who underwent laparoscopic surgery were retrospectively analyzed. Medical records (age, gender, body mass index (BMI), hypertension, diabetes mellitus, tumor location, preoperative laboratory test results, imaging results, complications) were collected. The relationship between PNI, NRI, CONUT and postoperative complications were analyzed.

**Results:**

There were 584 (41.6%) patients with postoperative complications and 821 (58.4%) without. The distribution of CONUT grade and the means PNI and NRI were significant differences between the two groups (all *p<*0.001). When postoperative complication was set as the endpoint of PNI and NRI, the cutoff value of PNI and NRI was 43.425 and 100.045, respectively. Logistic regression analysis showed that low NRI level (<100.045 vs. ≥100.045) (odds ratio (OR): 1.863, 95% confidence interval (CI): 1.377-2.522, *p* < 0.001), abnormal CONUT score (OR: 1.333, 95% CI: 1.002-1.772, *p* = 0.048) were significantly associated with postoperative complications.

**Conclusions:**

Low NRI level, abnormal CONUT score, underweight, and intestinal tumor were significantly associated with postoperative complications in gastrointestinal tumors patients with aged ≤ 60 who performed laparoscopic surgery.

## 1 Introduction

Gastrointestinal tumors are a general term for a large category of malignant tumors occurring in the gastrointestinal tract, mainly including gastric tumors and intestinal tumors (1, 2). These tumors originate from the mucosal epithelial cells of the gastrointestinal tract. Under the combined effect of various genetic, environmental and lifestyle factors, the cells undergo abnormal proliferation and disordered differentiation, and then form malignant lesions that are invasive and metastatic (3, 4). In recent years, the prevalence of gastrointestinal tumors has shown a significant upward trend worldwide (5). According to the latest global cancer statistics, the incidence rates of colorectal cancer and gastric cancer rank third and fifth among global cancers respectively (6). From the perspective of geographical distribution, the incidence of gastrointestinal tumors is particularly prominent in East Asia, Eastern Europe, and some developing countries (7). Gastrointestinal tumors are common malignant tumors in China, with a rather severe incidence situation, presenting characteristics such as a high incidence rate and an obvious trend of younger age (8, 9).

At present, the treatment methods for gastrointestinal tumors are diversified, mainly including surgical treatment, chemotherapy, radiotherapy, targeted therapy, and immunotherapy, and so on (10–12). Surgical treatment, as the main radical approach for gastrointestinal tumors, strives for a cure for patients by removing the tumor and the surrounding tissues that may be invaded (13, 14). In the field of surgical treatment, the application of laparoscopic technology has brought revolutionary changes to the treatment of gastrointestinal tumors (15). Laparoscopic surgery involves creating tiny operating holes in the patient’s abdomen, placing laparoscopes and surgical instruments into the abdominal cavity, and doctors perform the operation with the help of a high-definition imaging system (16). Compared with traditional open surgery, laparoscopic surgery has less trauma, less postoperative pain, significantly faster recovery speed of patients, can effectively shorten the hospital stay, and reduce the incidence of postoperative complications (17, 18). At present, laparoscopic surgery has been widely used in the treatment of early gastric cancer and colorectal cancer, and is gradually expanding to middle and advanced cases, playing an increasingly important role in improving the quality of life and long-term survival rate of patients (19, 20).

Although laparoscopic techniques have shown significant advantages in the treatment of gastrointestinal tumors, there is still an inevitable risk of certain complications (21, 22). The common complications of laparoscopic treatment for gastrointestinal tumors mainly include surgery-related complications and complications during the postoperative recovery stage. During the surgical process, vascular injury and organ perforation may occur, which are related to the location of the tumor and the complexity of the anatomical structure (23). During the postoperative recovery stage, patients may face complications such as incision infection, anastomotic leakage, and intestinal obstruction, which not only prolong the hospital stay but also may affect the rehabilitation process and long-term prognosis of the patients (24).

The occurrence of complications in laparoscopic treatment of gastrointestinal tumors is comprehensively influenced by multiple factors. Patients with intestinal tumors are prone to malnutrition due to the consumption of the tumors themselves, eating disorders and metabolic disorders related to the tumors (25). Malnutrition not only leads to a decline in the immune function of patients, weakening their stress response to surgical trauma, but also affects the ability of tissue repair and healing, making patients face a higher risk of complications after laparoscopic treatment (26). Prognostic nutritional index (PNI) is a quantitative indicator that assesses the nutritional status and prognosis of surgical patients through serum albumin levels and peripheral blood lymphocyte counts (27, 28). Nutritional risk index (NRI) is a scoring system that assesses the nutritional status and prognosis of patients based on serum albumin levels and the ratio of actual weight to ideal weight (29, 30). The Controlled Nutritional Status (CONUT) score is a nutritional status assessment tool that combines serum albumin, lymphocyte count and total cholesterol to reflect immune nutrition indicators (31). These three indicators are all quantitatively evaluated by integrating serological indicators, weight changes, or laboratory test results. These indicators not only avoid the limitation of single indicator being susceptible to non-nutritional factors, but also can more comprehensively and accurately reflect the nutritional status of patients. Moreover, they are easy to calculate and the data are easy to obtain. They are potential valuable indicators in disease diagnosis and treatment assessment.

Compared with the elderly population, non-elderly patients with gastrointestinal tumors have differences in common clinical symptoms, are more aggressive in tumor biology, and are more likely to be diagnosed at an advanced stage (32). Early-onset gastrointestinal tumors and late-onset gastrointestinal tumors have significant differences in molecular characteristics and clinical manifestations (33, 34). There are few research reports on the risk assessment of postoperative complications of gastrointestinal tumors in non-elderly people. There are few studies on the relationship of PNI, NRI and CONUT and occurrence of complications after laparoscopic treatment in patients with gastrointestinal tumors aged ≤60 years. The purpose of this study is to investigate the relationship between them.

## 2 Materials and methods

### 2.1 Study cohort

This study was a retrospective cohort study. Patients with early-onset (≤60 years old) gastrointestinal tumors who underwent laparoscopic surgery at Meizhou People’s Hospital from October 2017 to April 2025 were continuously included. Inclusion criteria (1): diagnosed with primary gastrointestinal malignant tumors and the age of onset is ≤60 years old (2); underwent laparoscopic surgery for the first time; (3) the clinical data are complete, including preoperative examinations, surgical records, postoperative recovery and follow-up data. Exclusion criteria: (1) combined with other malignant tumors; (2) neoadjuvant chemoradiotherapy was received before the operation; (3) there are surgical contraindications such as severe cardiopulmonary dysfunction; (4) cases converted to open abdominal surgery.

**Figure 1:**
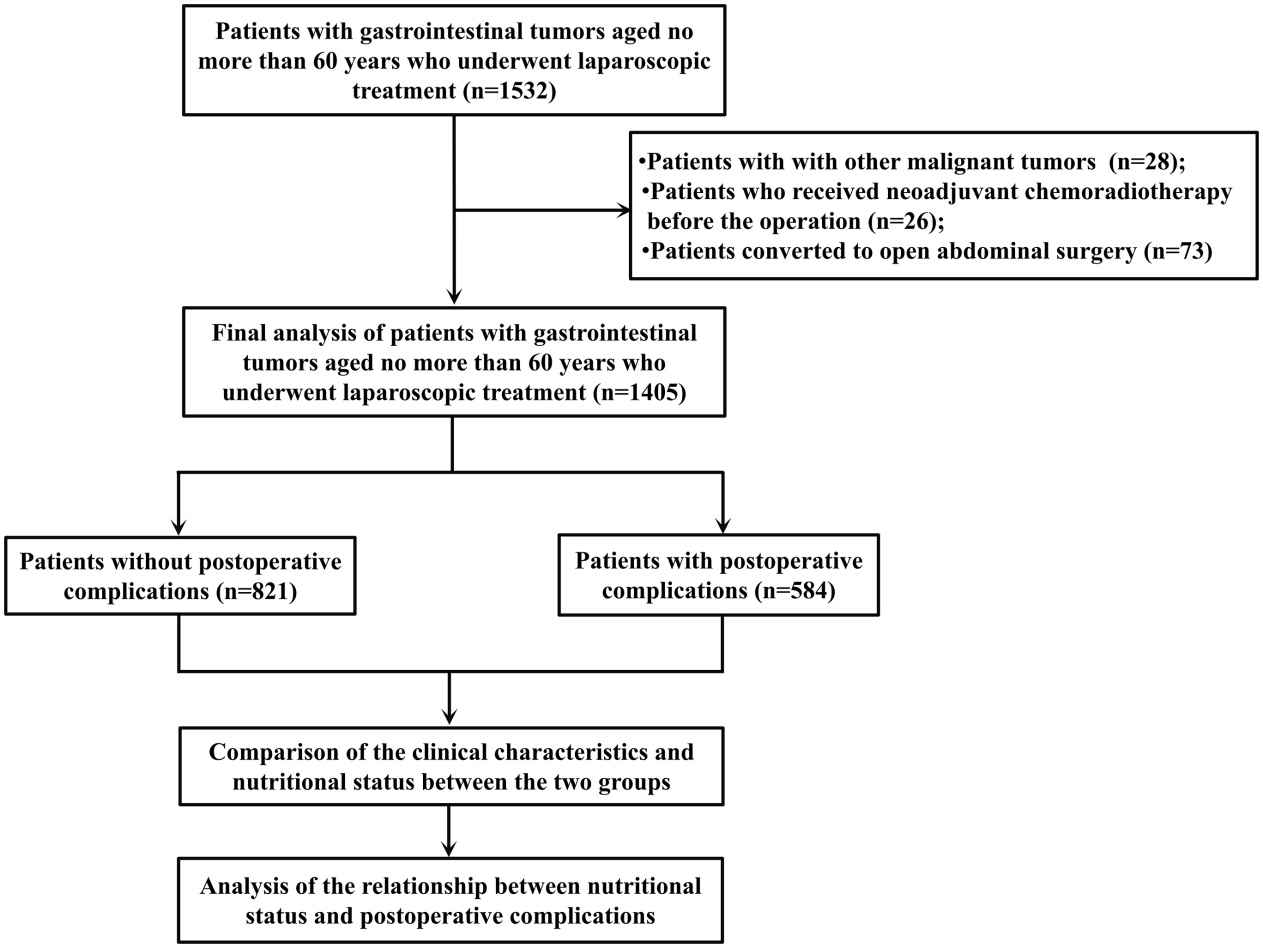
The flowchart of this study.

The sample size was calculated based on an observational study design, using the Power Analysis and Sample Size (PASS) Software. It focuses on the analysis of the associations between PNI, NRI, CONUT and postoperative complications of laparoscopic gastrointestinal tumors. Chi-square test (for univariate comparisons between groups) and logistic regression (for multivariate analysis) are used to set the statistical parameters: significance level α = 0.05 (two-sided test), power (1 - β) = 0.80 - 0.90. Based on clinical data, the overall postoperative complication rate is assumed to be 15%. The complication rate in the study group is hypothesized to be 15% - 20% higher than that in the control group. The sample is divided in a 1:1 ratio and 10% - 15% of the cases are reserved for loss to follow-up. For univariate comparisons, the total sample size needs to be 180–220 cases. If other confounding factors are included, the sample size needs to be increased by 30% - 40%. After calculation, at least 240–310 patients need to be included in the study. Ultimately, a total of 1405 patients who met the criteria were included. This study was approved by the Human Ethics Committees of the Meizhou People’s Hospital. The flowchart of present study is shown in **Figure 1**.

### 2.2 Data collection

In the hospital’s electronic medical record system, clinical data of patients are collected, including: age, gender, body mass index (BMI), hypertension, diabetes mellitus, tumor location (stomach and colorectal), preoperative laboratory test results (serum albumin, peripheral lymphocyte count, and total cholesterol level), imaging results, whether postoperative complications occurred and the types of complications (including bleeding, infection, anastomotic fistula, intestinal obstruction, intestinal adhesion, and poor wound healing, and so on). Postoperative complications are defined as any surgery-related adverse events that occur in patients within 30 days after the operation. The BMI was divided into three grades: underweight (<18.5 kg/m^2^), normal weight (18.5-23.9 kg/m^2^), and overweight (≥24.0 kg/m^2^) according to the standards of the Chinese population (35, 36).

### 2.3 Data processing and statistical analysis

The preoperative serum albumin concentration, peripheral lymphocyte count, and total cholesterol level of the patients were scored as follows: (1)the serum albumin: ①≥35g/L: 0 point, ②30-34.9g/L: 2 points, ③25-29.9g/L: 4 points, and ④<25g/L: 6 points; (2) peripheral lymphocyte count: ①≥1.6 × 10^9^ count/L: 0 point, ②1.2-1.59 × 10^9^ count/L: 1 point, ③0.8-1.19 × 10^9^ count/L: 2 points, and ④<0.8 × 10^9^ count/L: 3 points; (3) total cholesterol: ①>180mg/dL: 0 point, ②140-180mg/dL: 1 point, ③≥100 and <140mg/dL: 2 points, and ④<100mg/dL: 3 points. The CONUT score is the sum of the scores of three indicators: total lymphocyte count in peripheral blood, serum albumin and total cholesterol level. Degree of malnutrition based on the CONUT score: (1)normal: 0–1 point, (2) light: 2–4 points, (3) moderate: 5–8 points, and (4) severe: 9–12 points (37).

NRI=1.519×albumin (g/L) + 41.7×(current weight/IBW) [note: ideal body weight (IBW) = Height(m)^2^×22] (38).

PNI=Serum albumin + 5 × lymphocyte count.

### 2.4 Statistical analysis

Data analysis was performed using SPSS statistical software version 26.0 (IBM Inc., USA). Continuous data were compared using the Mann-Whitney U test. Categorical variables are expressed as the number of cases (%), and compared between groups using the χ^2^ test. The optimal thresholds of PNI and NRI were evaluated by receiver operating characteristic (ROC) curve analysis. Logistic regression analysis was used to analysis the relationship of PNI, NRI, CONUT and postoperative complications. *p* < 0.05.

## 3 Results

### 3.1 Clinical features of the gastrointestinal tumors patients with aged ≤60 who performed laparoscopic surgery

A total of 792 male patients (56.4%) and 613 female patients (43.6%) were enrolled. Regarding BMI, 136 patients (9.7%) were underweight, 744 (53.0%) had normal weight, and 525 (37.4%) were overweight. The number of patients with a history of smoking, history of alcohol drinking, hypertension, and diabetes mellitus was 136 (9.7%), 69 (4.9%), 185 (13.2%), and 126 (9.0%), respectively. Tumor types included colorectal cancer (1040 cases, 74.0%), gastric cancer (361 cases, 25.7%), and combined intestinal and gastric tumors (4 cases, 0.3%). There were 446 (31.7%), 753 (53.6%), 186 (13.2%), and 20 (1.4%) patients with CONUT normal, light, moderate, and severe grade. Postoperative complications occurred in 584 cases (41.6%) among all included patients (**Table 1**).

**Table 1:** The clinical features of the gastrointestinal tumors patients with aged ≤60 who performed laparoscopic surgery.

Clinical characteristics	Gastrointestinal tumors patients (n=1405)
Gender	
Male, n(%)	792 (56.4%)
Female, n(%)	613 (43.6%)
BMI (kg/m^2^)	
Underweight, n (%)	136 (9.7%)
Normal weight, n (%)	744 (53.0%)
Overweight, n (%)	525 (37.4%)
Cigarette smoking	
No, n(%)	1269 (90.3%)
Yes, n(%)	136 (9.7%)
Alcohol drinking	
No, n(%)	1336 (95.1%)
Yes, n(%)	69 (4.9%)
Hypertension	
No, n(%)	1220 (86.8%)
Yes, n(%)	185 (13.2%)
Diabetes mellitus	
No, n(%)	1279 (91.0%)
Yes, n(%)	126 (9.0%)
Tumor location	
Intestine, n(%)	1040 (74.0%)
Stomach, n(%)	361 (25.7%)
Intestine and stomach, n (%)	4 (0.3%)
Postoperative complications	
No, n(%)	821 (58.4%)
Yes, n(%)	584 (41.6%)
PNI, median (IQR)	44.30 (40.98, 48.50)
NRI, median (IQR)	101.29 (94.48, 107.70)
CONUT grades	
Normal, n(%)	446 (31.7%)
Mild, n(%)	753 (53.6%)
Moderate, n(%)	186 (13.2%)
Severe, n(%)	20 (1.4%)

BMI, body mass index; PNI, prognostic nutritional index; NRI, nutritional risk index; CONUT, controlling nutritional status; IQR, interquartile range.

### 3.2 Comparison of the clinical characteristics of patients with and without postoperative complication

Compared with patients without postoperative complications, those with postoperative complications had significantly higher proportions of underweight (15.4% vs. 5.6%, *p* < 0.001), cigarette smoking (11.8% vs. 8.2%, *p* = 0.028), alcohol drinking (6.8% vs. 3.5%, *p* = 0.006), and intestinal tumor (80.0% vs. 69.8%, *p* < 0.001). The mean PNI (*p* < 0.001) and NRI (*p* < 0.001) of patients with postoperative complications were lower than those without postoperative complications. A statistically significant difference was observed in the distribution of CONUT grades between the two groups (*p* < 0.001). There was no statistically significant difference in age, hypertension, and diabetes mellitus between the two groups (**Table 2**).

**Table 2:** Comparison of the clinical characteristics of patients with and without postoperative complications.

Clinical characteristics	Patients without postoperative complications (n=821)	Patients with postoperative complications (n=584)	*p* (χ^2^/Z)
Gender			
Male, n(%)	456 (55.5%)	336 (57.5%)	0.478 (χ^2^ = 0.551)
Female, n(%)	365 (44.5%)	248 (42.5%)	
BMI (kg/m^2^)			
Underweight, n (%)	46 (5.6%)	90 (15.4%)	<0.001 (χ^2^ = 38.788)
Normal weight, n (%)	464 (56.5%)	280 (47.9%)	
Overweight, n (%)	311 (37.9%)	214 (36.6%)	
Cigarette smoking			
No, n(%)	754 (91.8%)	515 (88.2%)	0.028 (χ^2^ = 5.212)
Yes, n(%)	67 (8.2%)	69 (11.8%)	
Alcohol drinking			
No, n(%)	792 (96.5%)	544 (93.2%)	0.006 (χ^2^ = 8.040)
Yes, n(%)	29 (3.5%)	40 (6.8%)	
Hypertension			
No, n(%)	711 (86.6%)	509 (87.2%)	0.810 (χ^2^ = 0.092)
Yes, n(%)	110 (13.4%)	75 (12.8%)	
Diabetes mellitus			
No, n(%)	748 (91.1%)	531 (90.9%)	0.925 (χ^2^ = 0.014)
Yes, n(%)	73 (8.9%)	53 (9.1%)	
Tumor location			
Intestine, n(%)	573 (69.8%)	467 (80.0%)	<0.001 (χ^2^ = 18.901)
Stomach, n(%)	246 (30.0%)	115 (19.7%)	
Intestine and stomach, n (%)	2 (0.2%)	2 (0.3%)	
PNI, median (IQR)	45.20 (42.05, 49.20)	43.00 (39.40, 47.44)	<0.001 (Z=-7.322)
NRI, median (IQR)	102.82 (96.78, 108.42)	99.08 (91.55, 106.42)	<0.001 (Z=-6.853)
CONUT			
Normal, n(%)	303 (36.9%)	143 (24.5%)	<0.001 (χ^2^ = 61.959)
Light, n(%)	443 (54.0%)	310 (53.1%)	
Moderate, n(%)	72 (8.8%)	114 (19.5%)	
Severe, n(%)	3 (0.4%)	17 (2.9%)	

BMI, body mass index; PNI, prognostic nutritional index; NRI, nutritional risk index; CONUT, controlling nutritional status; IQR, interquartile range.

### 3.3 Comparison of the clinical characteristics of patients with and without postoperative complication in patients with intestinal tumor

Among patients with intestinal tumors, those who developed postoperative complications had significantly higher proportions of underweight (14.6% vs. 4.9%, *p* < 0.001), alcohol drinking history (5.4% vs. 2.6%, *p* = 0.024), and abnormal CONUT score (74.1% vs. 63.7%, *p* < 0.001) compared to those without complications. Additionally, the complication group exhibited lower mean PNI (*p* < 0.001) and NRI (*p* < 0.001) (**Table 3**).

**Table 3:** Comparison of the clinical characteristics of patients with and without postoperative complications in patients with intestinal tumor.

Clinical characteristics	Patients with intestinal tumor (n=1040)	Patients without postoperative complications (n=573)	Patients with postoperative complications (n=467)	*p* (χ^2^/Z)
Gender				
Male, n(%)	579 (55.7%)	316 (55.1%)	263 (56.3%)	0.707 (χ^2^ = 0.142)
Female, n(%)	461 (44.3%)	257 (44.9%)	204 (43.7%)	
BMI (kg/m^2^)				
Underweight, n (%)	96 (9.2%)	28 (4.9%)	68 (14.6%)	<0.001 (χ^2^ = 28.934)
Normal weight, n (%)	526 (50.6%)	307 (53.6%)	219 (46.9%)	
Overweight, n (%)	418 (40.2%)	238 (41.5%)	180 (38.5%)	
Cigarette smoking				
No, n(%)	955 (91.8%)	535 (93.4%)	420 (89.9%)	0.053 (χ^2^ = 4.039)
Yes, n(%)	85 (8.2%)	38 (6.6%)	47 (10.1%)	
Alcohol drinking				
No, n(%)	1000 (96.2%)	558 (97.4%)	442 (94.6%)	0.024 (χ^2^ = 5.206)
Yes, n(%)	40 (3.8%)	15 (2.6%)	25 (5.4%)	
Hypertension				
No, n(%)	897 (86.3%)	491 (85.7%)	406 (86.9%)	0.588 (χ^2^ = 0.338)
Yes, n(%)	143 (13.8%)	82 (14.3%)	61 (13.1%)	
Diabetes mellitus				
No, n(%)	947 (91.1%)	523 (91.3%)	424 (90.8%)	0.827 (χ^2^ = 0.073)
Yes, n(%)	93 (8.9%)	50 (8.7%)	43 (9.2%)	
PNI, median (IQR)	44.30 (40.81, 48.49)	45.30 (42.00, 49.23)	43.00 (39.40, 47.50)	<0.001 (Z=-6.235)
NRI, median (IQR)	102.03 (94.47, 108.10)	103.53 (97.37, 108.91)	99.13 (91.65, 106.82)	<0.001 (Z=-6.493)
CONUT				
Normal, n(%)	329 (31.6%)	208 (36.3%)	121 (25.9%)	<0.001 (χ^2^ = 12.843)
Abnormal, n(%)	711 (68.4%)	365 (63.7%)	346 (74.1%)	

BMI, body mass index; PNI, prognostic nutritional index; NRI, nutritional risk index; CONUT, controlling nutritional status; IQR, interquartile range.

### 3.4 Comparison of the clinical characteristics of patients with and without postoperative complication in patients with gastric tumor

Among patients with gastric tumors, those who experienced postoperative complications had significantly higher proportions of underweight (18.3% vs. 7.3%, *p* = 0.005), alcohol drinking history (13.0% vs. 5.7%, *p* = 0.022), and abnormal CONUT score (80.9% vs. 61.4%, *p* < 0.001) compared with those without complications. Meanwhile, the mean values of PNI (*p* < 0.001) and NRI (*p* = 0.006) were significantly lower in the complication group (**Table 4**).

**Table 4:** Comparison of the clinical characteristics of patients with and without postoperative complications in patients with gastric tumor.

Clinical characteristics	Patients with gastric tumor (n=361)	Patients without postoperative complications (n=246)	Patients with postoperative complications (n=115)	*p* (χ^2^/Z)
Gender				
Male, n(%)	210 (58.2%)	138 (56.1%)	72 (62.6%)	0.254 (χ^2^ = 1.365)
Female, n(%)	151 (41.8%)	108 (43.9%)	43 (37.4%)	
BMI (kg/m^2^)				
Underweight, n (%)	39 (10.8%)	18 (7.3%)	21 (18.3%)	0.005 (χ^2^ = 10.345)
Normal weight, n (%)	216 (59.8%)	156 (63.4%)	60 (52.2%)	
Overweight, n (%)	106 (29.4%)	72 (29.3%)	34 (29.6%)	
Cigarette smoking				
No, n(%)	310 (85.9%)	217 (88.2%)	93 (80.9%)	0.074 (χ^2^ = 3.482)
Yes, n(%)	51 (14.1%)	29 (11.8%)	22 (19.1%)	
Alcohol drinking				
No, n(%)	332 (92.0%)	232 (94.3%)	100 (87.0%)	0.022 (χ^2^ = 5.734)
Yes, n(%)	29 (8.0%)	14 (5.7%)	15 (13.0%)	
Hypertension				
No, n(%)	320 (88.6%)	219 (89.0%)	101 (87.8%)	0.859 (χ^2^ = 0.112)
Yes, n(%)	41 (11.4%)	27 (11.0%)	14 (12.2%)	
Diabetes mellitus				
No, n(%)	328 (90.9%)	223 (90.7%)	105 (91.3%)	0.849 (χ^2^ = 0.040)
Yes, n(%)	33 (9.1%)	23 (9.3%)	10 (8.7%)	
PNI, median (IQR)	44.45 (41.40, 48.60)	45.05 (42.38, 49.25)	43.25 (39.80, 47.15)	<0.001 (Z=-3.697)
NRI, median (IQR)	100.10 (94.59, 105.96)	100.87 (96.23, 106.63)	98.90 (91.16, 104.93)	0.006 (Z=-2.774)
CONUT				
Normal, n(%)	117 (32.4%)	95 (38.6%)	22 (19.1%)	<0.001 (χ^2^ = 13.585)
Abnormal, n(%)	244 (67.6%)	151 (61.4%)	93 (80.9%)	

BMI, body mass index; PNI, prognostic nutritional index; NRI, nutritional risk index; CONUT, controlling nutritional status; IQR, interquartile range.

### 3.5 Logistic regression analysis of risk factors of postoperative complications in gastrointestinal tumors patients with aged ≤60 who performed laparoscopic surgery

**Figure 2:**
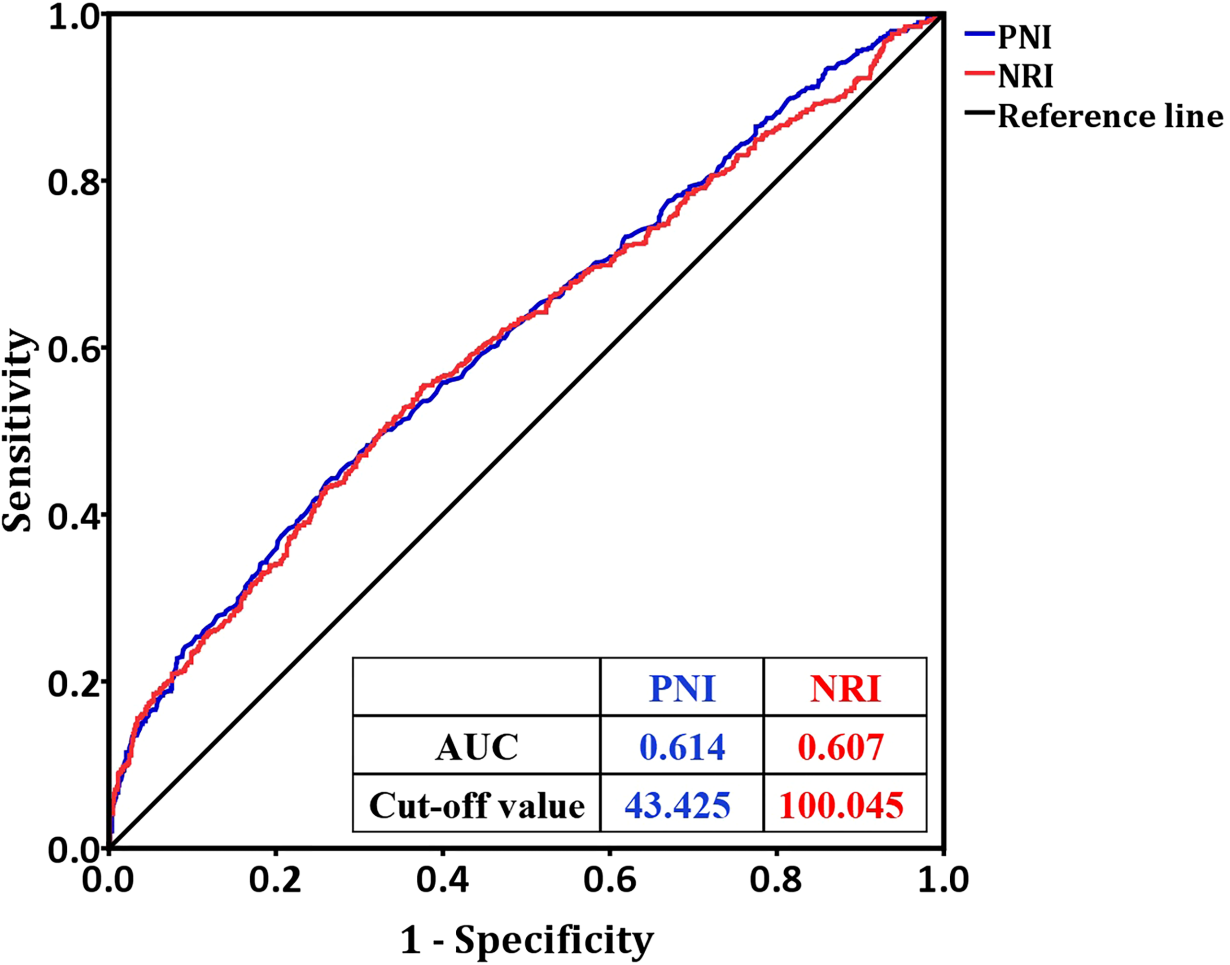
The ROC curve analysis of PNI and NRI to distinguish postoperative complications in gastrointestinal tumors patients with aged ≤60 who performed laparoscopic surgery. PNI, prognostic nutritional index; NRI, nutritional risk index.

When postoperative complication was designated as the endpoint for analyzing PNI and NRI, ROC curve analysis revealed that the optimal cutoff value of PNI was 43.425, with a sensitivity of 52.6%, specificity of 63.5%, and area under the ROC curve (AUC) of 0.614. For NRI, the cutoff value was 100.045, which yielded a sensitivity of 55.5%, specificity of 62.2%, and AUC of 0.607 (**Figures 1**, **2**).

Univariate analysis results demonstrated that reduced PNI level (<43.425 vs. ≥43.425) (odds ratio (OR): 1.925, 95% confidence interval (CI): 1.551-2.38, *p* < 0.001) and NRI level (<100.045 vs. ≥100.045) (OR: 2.054, 95% CI: 1.656-2.549, *p* < 0.001), abnormal CONUT score (OR: 1.804, 95% CI: 1.425-2.284, *p* < 0.001), underweight (OR: 3.242, 95% CI: 2.206-4.765, *p* < 0.001), cigarette smoking (OR: 1.508, 95% CI: 1.058-2.149, *p* = 0.023), alcohol drinking (OR: 2.008, 95% CI: 1.230-3.279, *p* = 0.005), and intestinal tumor (OR: 1.743, 95% CI: 1.354-2.245, *p* < 0.001) were significantly associated with postoperative complications (**Table 5**). In multivariate logistic regression analysis, reduced NRI level (<100.045 vs. ≥100.045) (OR: 1.863, 95% CI: 1.377-2.522, *p* < 0.001), abnormal CONUT score (OR: 1.333, 95% CI: 1.002-1.772, *p* = 0.048), underweight (OR: 2.764, 95% CI: 1.846-4.139, *p* < 0.001), and intestinal tumor (OR: 1.906, 95% CI: 1.459-2.490, *p* < 0.001) remained significantly associated with postoperative complications (**Table 5**).

**Table 5:** Logistic regression analysis of risk factors of postoperative complications in gastrointestinal tumors patients with aged ≤60 who performed laparoscopic surgery.

Variables	Unadjusted values		Adjusted values	
	OR (95% CI)	*p* values	Adjusted OR (95% CI)	*p* values
PNI (<43.425 vs. ≥43.425)	1.925 (1.551-2.388)	<0.001	1.232 (0.917-1.654)	0.167
NRI (<100.045 vs. ≥100.045)	2.054 (1.656-2.549)	<0.001	1.863 (1.377-2.522)	<0.001
CONUT (abnormal vs. normal)	1.804 (1.425-2.284)	<0.001	1.333 (1.002-1.772)	0.048
Gender (male vs. female)	1.084 (0.875-1.343)	0.458	1.069 (0.884-1.353)	0.581
BMI (kg/m^2^)				
Normal weight	1.000 (reference)	–	1.000 (reference)	–
Underweight	3.242 (2.206-4.765)	<0.001	2.764 (1.846-4.139)	<0.001
Overweight	1.140 (0.907-1.433)	0.261	1.496 (1.137-1.969)	0.104
Cigarette smoking (yes vs. no)	1.508 (1.058-2.149)	0.023	1.277 (0.803-2.029)	0.302
Alcohol drinking (yes vs. no)	2.008 (1.230-3.279)	0.005	1.833 (0.989-3.399)	0.054
Hypertension (yes vs. no)	0.952 (0.695-1.305)	0.761	1.071 (0.761-1.507)	0.694
Diabetes mellitus (yes vs. no)	1.023 (0.706-1.482)	0.905	1.203 (0.810-1.787)	0.359
Tumor location (intestine vs. stomach)	1.743 (1.354-2.245)	<0.001	1.906 (1.459-2.490)	<0.001

PNI, prognostic nutritional index; NRI, nutritional risk index; CONUT, controlling nutritional status; BMI, body mass index; OR, odds ratio; CI, confidence interval.

In patients with intestinal tumor, reduced NRI level (OR: 2.653, 95% CI: 1.962-3.587, *p* < 0.001), abnormal CONUT score (OR: 1.529, 95% CI: 1.163-2.011, *p* = 0.002), underweight (OR: 2.801, 95% CI: 1.716-4.572, *p* < 0.001) were significantly associated with postoperative complications. In patients with gastric tumor, reduced NRI level (OR: 1.772, 95% CI: 1.048-2.994, *p* = 0.033), abnormal CONUT score (OR: 2.226, 95% CI: 1.209-4.098, *p* = 0.010), underweight (OR: 2.864, 95% CI: 1.363-6.020, *p* = 0.005) were significantly associated with postoperative complications (**Table 6**).

**Table 6:** Logistic regression analysis of risk factors of postoperative complications in patients with intestinal tumor and gastric tumor, respectively.

Variables	Patients with intestinal tumor				Patients with gastric tumor			
	Unadjusted values		Adjusted values		Unadjusted values		Adjusted values	
	OR (95% CI)	*p* values	Adjusted OR (95% CI)	*p* values	OR (95% CI)	*p* values	Adjusted OR (95% CI)	*p* values
PNI (<43.425 vs. ≥43.425)	1.912 (1.491-2.451)	<0.001	1.207 (0.854-1.705)	0.286	1.893 (1.207-2.968)	0.005	1.238 (0.689-2.223)	0.475
NRI (<100.045 vs. ≥100.045)	2.391 (1.859-3.074)	<0.001	2.653 (1.962-3.587)	<0.001	1.505 (0.964-2.351)	0.072	1.772 (1.048-2.994)	0.033
CONUT (abnormal vs. normal)	1.630 (1.246-2.130)	<0.001	1.529 (1.163-2.011)	0.002	2.660 (1.564-4.522)	<0.001	2.226 (1.209-4.098)	0.010
Gender (male vs. female)	1.084 (0.875-1.343)	0.458	1.044 (0.798-1.364)	0.755	1.310 (0.832-2.063)	0.243	1.135 (0.680-1.893)	0.628
BMI (kg/m^2^)								
Normal weight	1.000 (reference)	–	1.000 (reference)	–	1.000 (reference)	–	1.000 (reference)	–
Underweight	3.404 (2.121-5.464)	<0.001	2.801 (1.716-4.572)	<0.001	3.033 (1.512-6.087)	0.002	2.864 (1.363-6.020)	0.005
Overweight	1.060 (0.818-1.375)	0.659	1.611 (1.177-2.205)	0.003	1.228 (0.741-2.034)	0.426	1.218 (0.674-2.202)	0.513
Cigarette smoking (yes vs. no)	1.576 (1.008-2.462)	0.046	1.286 (0.732-2.258)	0.381	1.770 (0.967-3.242)	0.064	1.299 (0.568-2.971)	0.535
Alcohol drinking (yes vs. no)	2.104 (1.096-4.039)	0.025	1.714 (0.773-3.798)	0.185	2.486 (1.157-5.343)	0.020	2.131 (0.791-5.745)	0.135
Hypertension (yes vs. no)	0.900 (0.630-1.285)	0.561	0.980 (0.669-1.435)	0.917	1.124 (0.566-2.235)	0.738	1.424 (0.654-3.101)	0.373
Diabetes mellitus (yes vs. no)	1.061 (0.692-1.626)	0.787	1.272 (0.810-1.999)	0.297	0.923 (0.424-2.010)	0.841	1.028 (0.440-2.404)	0.948

PNI, prognostic nutritional index; NRI, nutritional risk index; CONUT, controlling nutritional status; BMI, body mass index; OR, odds ratio; CI, confidence interval.

## 4 Discussion

The analysis of patients undergoing laparoscopic surgery for early-onset gastrointestinal tumors, clarifying the risk factors related to the occurrence of postoperative complications is of great significance for optimizing perioperative management and reducing the incidence of complications. In this study, low NRI level, abnormal CONUT score, underweight, and intestinal tumor were significantly associated with postoperative complications in gastrointestinal tumors patients with aged ≤60 who performed laparoscopic surgery.

Low NRI level and abnormal CONUT score reflect the presence of varying degrees of malnutrition in patients before the operation (39). NRI assesses nutritional status through changes in serum protein levels and body weight (40). The CONUT score is comprehensively judged in combination with indicators such as serum protein, blood lipid and lymphocyte count (41). Previous studies found that the preoperative CONUT score may be an independent risk factor for postoperative complications in patients undergoing laparoscopic-assisted radical gastrectomy for gastric cancer (42, 43).

Compared with elderly patients, people aged ≤60 often neglect nutritional management due to their relatively good physical condition, or have irregular diets due to the fast pace of work and life, which leads to the failure to correct preoperative malnutrition in time (44). Malnutrition can weaken the body’s immune defense ability and affect the protein synthesis and cell proliferation required during the wound healing process (45–47). For example, low serum albumin level lead to a decrease in plasma colloid osmotic pressure, which is prone to cause tissue edema and delay incision healing (48). The reduction in the number and impaired function of lymphocytes lead to a decline in the body’s resistance to pathogens such as bacteria and viruses, increasing the risk of postoperative infection (49). The results of this study are consistent with the conclusion of multiple studies indicating that malnutrition is an independent risk factor for postoperative complications (50–53). It suggests that preoperative nutritional assessment should be emphasized in clinical practice, and preoperative nutritional support should be provided when necessary to improve the nutritional status of patients and reduce the incidence of postoperative complications.

Patients with underweight often have a long-term situation of insufficient energy intake or excessive energy consumption. Patients with underweight have less stored glycogen, fat and protein in their bodies, which cannot provide sufficient energy and material basis for postoperative body repair and stress response (54, 55). In addition, being underweight is often accompanied by a reduction in muscle mass, which leads to a slow recovery of the patient’s physical functions after surgery, a decline in the patient’s ability to expel phlegm independently such as coughing and expectoration, and an increased risk of respiratory complications such as pulmonary infection and atelectasis (56). For such patients, a personalized nutritional supplementation plan should be formulated during the perioperative period to promote the recovery of the body.

Compared with gastric tumors, intestinal tumors are more significantly associated with postoperative complications, which may be related to the physiological characteristics of the intestine. The intestinal function of young patients is relatively active (57). During the recovery process of intestinal peristalsis after surgery, if the anastomosis does not heal well, it is easy to cause anastomotic leakage due to the tension generated by peristalsis (58). The intestinal contents are rich in bacteria, and the postoperative intestinal anastomosis faces a higher risk of infection (59). Meanwhile, the recovery of intestinal peristaltic function is relatively slow, and complications such as intestinal obstruction and intestinal adhesion are prone to occur (60). In addition, intestinal tumor surgery may require complex operations such as intestinal fistula, further increasing the probability of surgical trauma and postoperative complications (61). Therefore, for patients with intestinal tumors, more attention should be paid to the protection of the intestine and anastomosis techniques during the operation, and the management of intestinal function recovery should be strengthened after the operation.

In this study, poor nutritional status based on CONUT score and NRI may be related to the occurrence of complications after laparoscopic treatment in patients with gastrointestinal tumors aged ≤60 years. It provides important reference data for optimizing the clinical diagnosis and treatment strategies for this disease population. First, clinicians can incorporate CONUT and NRI into routine assessments and establish a comprehensive assessment model to enhance the early warning of complications. Second, based on the assessment results, implement stratified and precise preoperative nutritional intervention to improve patients’ nutritional reserves, reduce the risk of complications, guide intraoperative management, and formulate individualized rehabilitation and nutritional follow-up plans after surgery. Third, promote the integration of nutritional assessment into the treatment pathway and the multidisciplinary diagnosis and treatment (MDT) between surgery and nutrition departments to improve patient prognosis.

Although this study has obtained some valuable results, it has certain limitations. Firstly, this study is a single-center retrospective analysis. The representativeness of the sample is limited, which may affect the universality of the conclusion. Secondly, the influence of the differences in different age groups (such as youth and middle age) among patients aged ≤60 years on the outcome was not fully considered. Thirdly, in this study, the AUC values were relatively low. This might be due to the fact that the occurrence of postoperative gastrointestinal tumor complications is influenced by numerous factors, which has affected the discriminatory ability of this model. Finally, since the evolution of laparoscopic surgical techniques during the study period (such as optimization of the operation process, update of equipment, and improvement of the surgeon’s experience) was not fully considered, it may lead to interference from confounding factors in the analysis of the association between nutritional status and the risk of postoperative complications. Future studies can conduct multicenter and prospective cohort studies to expand the sample size and refine age stratification. At the same time, more influencing factors can be included to deeply explore the occurrence mechanism of postoperative complications in patients with gastrointestinal tumors aged ≤60 years, providing more precise prevention and treatment strategies for clinical practice.

## 5 Conclusion

Low NRI level, abnormal CONUT score, underweight, and intestinal tumor were significantly associated with postoperative complications in gastrointestinal tumors patients with aged ≤60 who performed laparoscopic surgery.

## Data Availability

The original contributions presented in the study are included in the article/supplementary material. Further inquiries can be directed to the corresponding author.
